# Highly sensitive determination of heavy metals in water prior to and after remediation using *Citrofortunella Microcarpa*

**DOI:** 10.1038/s41598-020-80672-9

**Published:** 2021-01-14

**Authors:** Shirley T. Palisoc, Remuel Isaac M. Vitto, Marissa G. Noel, Katja T. Palisoc, Michelle T. Natividad

**Affiliations:** 1 Condensed Matter Research Unit, CENSER, De La Salle University, 2401 Taft Ave, 922 Manila, Philippines; 2 Physics Department, De La Salle University, 2401 Taft Ave, 922 Manila, Philippines; 3 Chemistry Department, De La Salle University, 2401 Taft Ave, 922 Manila, Philippines

**Keywords:** Chemistry, Materials science, Nanoscience and technology

## Abstract

A highly sensitive bismuth/silver nanoparticles/Nafion-modified screen-printed graphene electrode was fabricated and was utilized for the detection of trace lead (Pb) concentrations in river water samples prior to and after remediation using calamansi (*Citrofortunella Microcarpa)* rinds in different forms viz., ground sun-dried, dry-ashed, food-grade pectin, fractionated pectin, and alcohol insoluble solids—extracted pectin. All these forms of pectin remediated Pb in the water samples. Hence, this novel method of using calamansi rinds in different forms is an effective method for the removal of lead in water. The electrode was characterized using scanning electron microscopy and energy dispersive x-ray spectrometry which confirmed the presence of the modifiers on the electrode surface. The limit of detection of 267.6 ppt and the strong linear relationship between the Pb concentration and the anodic current response (R^2^ = 0.999) were obtained under optimized experimental conditions and parameters.

## Introduction

The increasing number of industrial, as well as, anthropologic activities contribute heavily to disease-causing heavy metal pollution of water resources. In high doses, these heavy metals cause acute and chronic toxicities in humans^1^. Lead (Pb), even in trace amounts, can severely affect the mental and physical development in children below six years of age^2^. Acute and long-term exposure to lead can cause gonadal dysfunction in men, including depressed sperm counts^3–6^, preterm delivery of pregnant women and other female reproduction dysfunction^7,8^. Inorganic lead compounds are deemed carcinogenic to humans^9^. It leaks into the environment and gets carried up the food chain from aquatic sources. In oceans, the concentration of this metal ranges from 2 to 30 ppt^10^. On the average, rivers contain between 3 and 30 ppb. The World Health Organization (WHO) set a limit of 50 ppb for Pb in 1995, which was reduced to 10 ppb in 2010^11^.

The detrimental effects of heavy metals on human health necessitate the development of sensors and methods which are cost-effective, highly sensitive, thoroughly selective, and have the advantage of portability. The traditional methods of heavy metal detection are high-performance liquid chromatography (HPLC), coupled with electrochemical- or UV–Vis-detectors, atomic absorption spectroscopy (AAS), inductively coupled plasma mass spectroscopy (ICP-MS) electrothermal atomic absorption spectrometry, flame atomic absorption spectrometry, and wet chemical methods such as colorimetry, titrimetry, and gravimetry^12–24^. Most of these methods require expensive, state-of-the-art bulky equipment manned by trained staff which inhibits in situ measurements. This is remedied by the use of electrochemical methods like anodic stripping voltammetry (ASV) due to its high sensitivity, fast response, cost-effectivity, simpler approach, and ease of adaptability to be integrated into portable, disposable devices for the *in-situ* multi-element of heavy metals^12,19,22–24^.

Screen-printed electrodes which are employed in anodic stripping voltammetry consist of the working, reference and counter electrodes bundled into a single miniaturized instrument. They are ready to use and are suitable for in situ monitoring. Modification of the working electrode by nanomaterials has previously been shown to enhance the sensitivity, selectivity, and stability of the electrode^25–32^. The large surface area-to-volume ratio of nanomaterials, such as silver nanoparticles (AgNP), increases mass transport, decreases solution resistance, and improves signal-to-noise ratio which all contribute to the sensor’s low detection limit^33^.

The adverse effects of lead on man which can even be fatal make it imperative to find ways to remove it from drinking water. The current methods of lead remediation include different chemical treatments that produce toxic sludge that is not environmentally friendly and are difficult to dispose of^34^. To prevent this, substances from natural sources such as the rinds of citrus fruits can be utilized to remove the metals present in the water. The main component of the peels is pectin, the ionic bonding of which with divalent cations gives it the ability to act as binding sites for heavy metals^35^. Since they are waste products, the remediation is low-cost and environment-friendly.

In this study, a highly sensitive bismuth (Bi)/AgNP/Nafion-modified screen-printed graphene electrode (SPGE) was developed for the determination of Pb by anodic stripping voltammetry. The unique approach in this work is the use of a facile method in fabricating the modified SPGE and its application in the determination of trace lead concentration in river water samples prior to and after a novel method of water remediation using different forms of pectin derived from calamansi (*Citrofortunella Microcarpa)* rinds which are abundant in the Philippines.

## Methodology

### Materials and equipment

The materials and equipment utilized in this study are the same as in our previous work^33^. A Bosch SAE200 electronic balance, Biobase vacuum drying oven, Thermolyne Barnstead 48000 Furnace, Bandelin Sonorex sonicator, Pyrex Beakers, Brandtech Transferpette S Pipette, and Hanna Instruments mini magnetic stirrer HI190M were used in the study. A Palmsens4 potentiostat was utilized for all anodic stripping voltammetry measurements. AA-6300 Shimadzu atomic absorption spectrophotometer was used in the atomic absorption spectroscopy analysis. Wooden screen-printing frames, Tultex screen polyester mesh with 200 threads per inch (tpi), a Tulstrip green film used as a stencil, a polyurethane squeegee, a film adhesol, and a manual screen stretcher were all obtained from Tulco Screen Printing Supply Inc. (Quezon City, Philippines). A Silhouette Cameo 3 automated cutter from Sandhel Trading (Quezon City, Philippines) was utilized to precisely cut the stencil design of the electrodes. One (1)-mm thick 4′ × 8′ polyvinyl chloride (PVC) sheets and a 4-color 1-station rotary screen-printing press were procured from Q-Ri Technologies Inc. (Paranaque City, Philippines).

### Chemicals and reagents

The chemicals and reagents utilized in this study are the same as in our previous work^33^. Bismuth powder procured from Luoyang Tongrun Info Technology Co., Ltd. (Luoyang City, Henan, China), silver nanoparticles procured from Sigma-Aldrich, and Nafion solutions from Fuel Cell Earth were used in the study. Solutions used for the optimization of parameters and concentrations of modifiers were made using lead chloride powder procured from Sigma-Aldrich. Carbon graphene paste (C2171023D1) procured from Gwent Group Ltd. (Monmouthshire Wales, United Kingdom) was used for the working and counter electrodes. Silver-silver chloride (Ag/AgCl) Paste (C2130809D5 60:40) was also procured from Gwent Group Ltd. and was utilized as the reference electrode. A Bosny No.190 Clear Acrylic Spray was utilized as the insulating layer for the electrodes.

### Screen-printed graphene electrode fabrication

**Figure 1:**
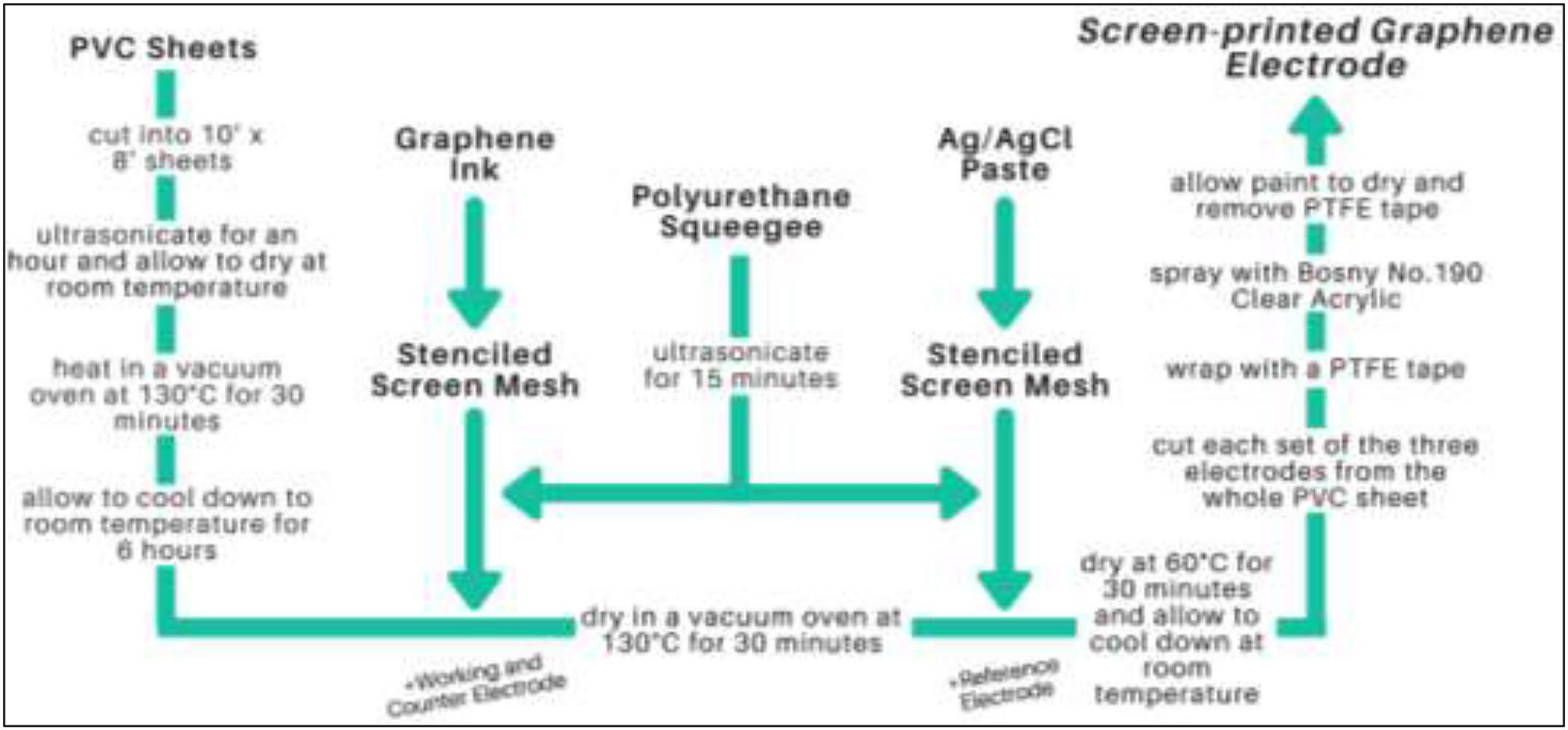
Schematic diagram of the SPGE fabrication.

The schematic diagram for the fabrication of the SPGE is shown in Fig. 1. Polyvinyl chloride sheets were cut into 10′ × 8′ sheets, ultrasonicated for an hour, and allowed to dry at room temperature. The PVC sheets were then heated in a vacuum oven at 130 °C for 30 min to avoid warping prior to screen printing the electrode and were then allowed to cool down to room temperature for 6 h. A polyurethane squeegee, ultrasonicated for 15 min, was used in printing the graphene ink through the stenciled screen mesh onto the PVC sheets to create the working and counter electrodes. The graphene ink was mixed thoroughly using a clean and sonicated PVC stick before pouring it into the screen mesh. The PVC sheets with the working and counter electrodes were then dried in a vacuum oven at 130 °C for 30 min. The reference electrodes were fabricated in the same manner. The only variation was the use of a silver-silver chloride (Ag/AgCl) paste. It was then dried again at 60 °C for 30 min and allowed to cool down at room temperature. Each set of the three electrodes were then individually cut from the whole PVC sheet and then wrapped with a polytetrafluoroethylene (PTFE) tape to cover the active surface of the working, counter, and reference electrodes, as well as the ends that will serve as connections to the potentiostat. Finally, they were sprayed with an insulating layer of Bosny No.190 Clear Acrylic and the PTFE tape was then removed. The final surface areas of the electrodes were as follows: (a) Working electrode: 0.5 cm^2^, (b) Counter electrode: 1.86 cm^2^, and (c) Reference electrode: 0.5 cm^2^. The approximate final electrode size was 7.4 cm × 2.5 cm.

### Fabrication of Bi/AgNP/nafion modified SPGEs

**Figure 2:**
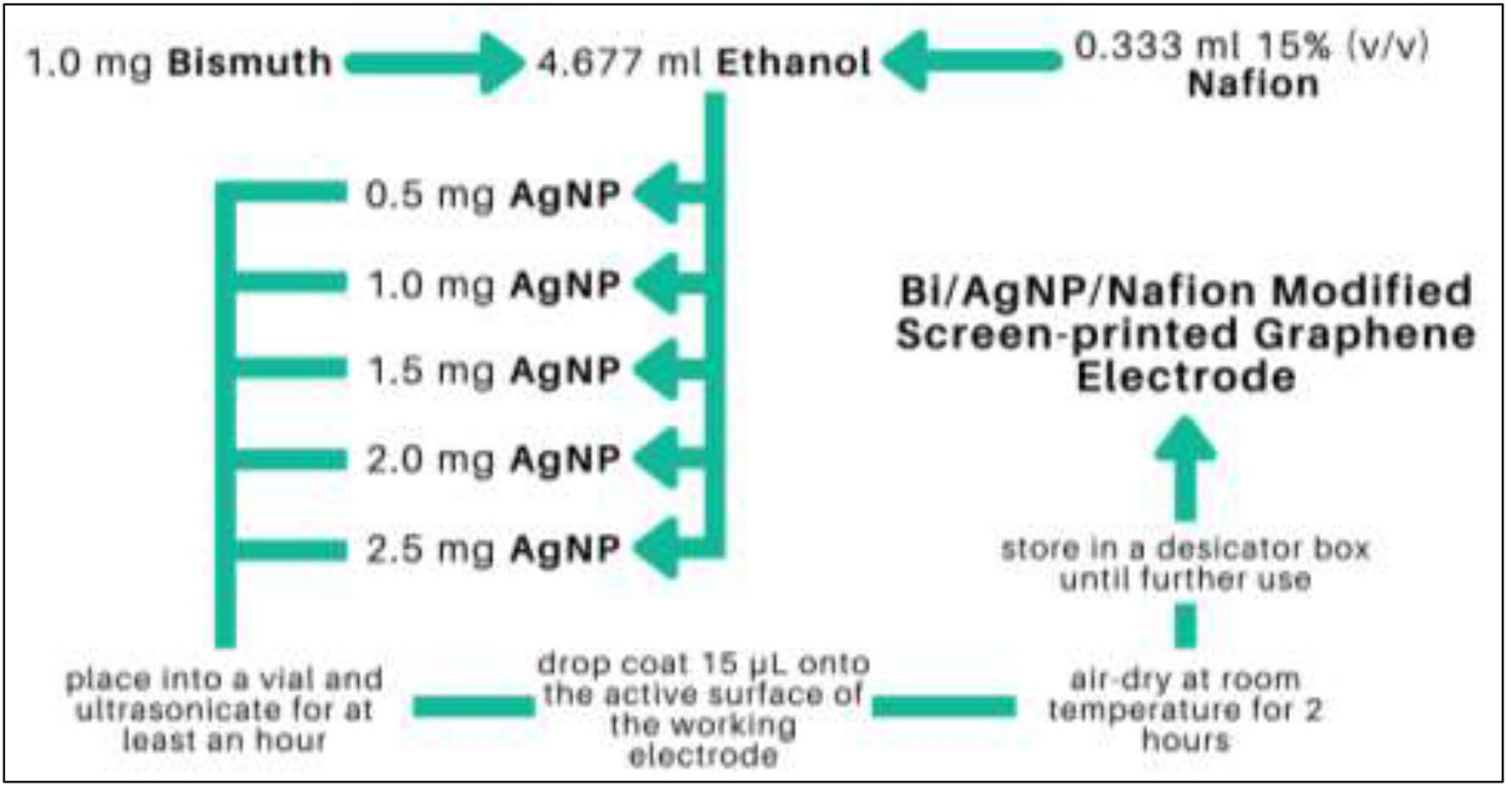
Schematic diagram for the modification of the SPGE.

The modified SPGE was fabricated using the procedure in our previous work^33^. The schematic diagram for the modification of the SPGE is shown in Fig. 2. Modification of the SPGE was done via the drop coating method by using a micropipette. The Bi concentration remained constant at 1.0 mg while the amount of AgNP was varied at 0.5 mg, 1.0 mg, 1.5 mg, 2.0 mg, and 2.5 mg. Each Bi-AgNP mixture was then incorporated into a 1% (v/v) Nafion solution consisting of 0.333 mL of 15% (v/v) Nafion and 4.667 mL ethanol. The resulting casting solutions were ultrasonicated for at least an hour before the application of 15 µL of the said solution onto the working electrode. The drop-coated electrodes were air-dried at room temperature for at least 2 h before use and were stored in a desiccator box.

### Preparation of the analyte solutions

Analyte solutions containing 10 ppm of Pb (1.3 mg of PbCl_2_) dissolved in 100 mL of deionized water were used for the determination of the optimum amount of AgNP as electrode modifier as well as the optimum ASV parameters. Analyte solutions prepared in the same manner were used in obtaining the calibration curve but with a further dilution to get the target concentrations of 30 ppb, 40 ppb, 50 ppb, 60 ppb, 100 ppb, 200 ppb, 300 ppb, 400 ppb, 500 ppb, and 600 ppb. All the resulting solutions were sonicated for 15 min before use.

### Preparation of the different forms of calamansi rinds

**Figure 3:**
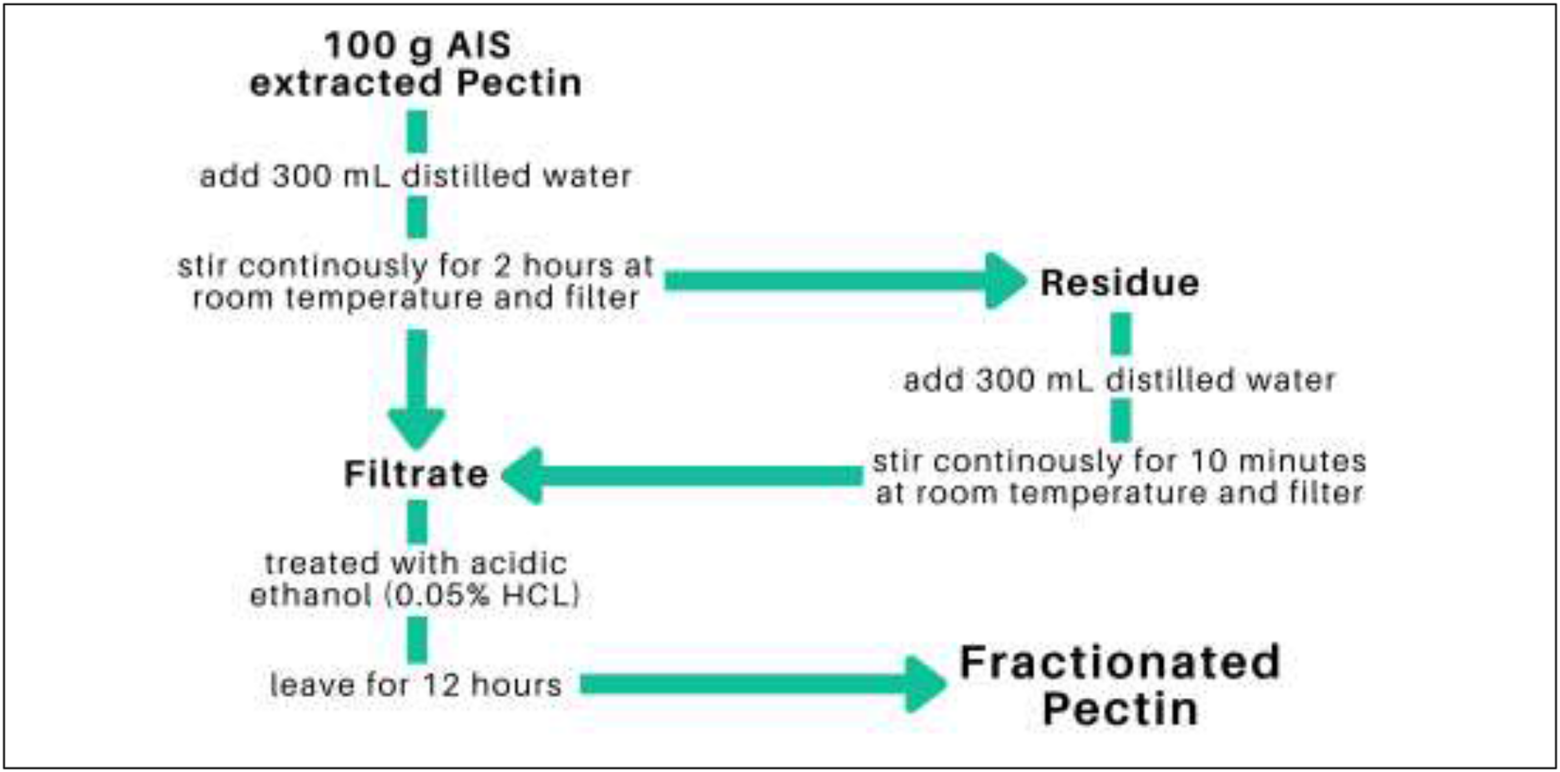
Schematic diagram for the fractionation of pectin.

The calamansi rinds were thoroughly washed and sun-dried for approximately 48 h and then stored at room temperature until further use. The sun-dried calamansi rinds were then ground with the use of a blender to produce a fine powder. The dry-ashed calamansi rinds were prepared by placing the ground sun-dried calamansi rinds in a crucible and heated to 500 °C for 6 h. Commercially available food-grade pectin was purchased from All About Baking, Quezon City, Metro Manila. The alcohol insoluble solids (AIS) extracted pectin was prepared by treating the calamansi rinds with 95% ethanol (preheated to 70 °C). The mixture was kept at 70 °C for 15 min and then cooled prior to extraction in a blender. The mixture was then filtered under suction to separate the insoluble material which was subsequently washed with warm 70% ethanol followed by 95% ethanol (at 70 °C). The alcohol–insoluble solids were freeze-dried and then ground (to pass through a 60—mesh sieve) and stored at room temperature until further use. The schematic diagram for the fractionation of pectin is shown in Fig. 3.

### Remediation of Pasig River water samples

The modified electrode was utilized in the determination of Pb concentrations in water samples obtained from Pasig River before and after the remediation process. The water samples were remediated using different forms of calamansi namely, ground sun-dried, dry-ashed, food-grade pectin, fractionated pectin, and AIS extracted pectin. The water samples were heat-treated prior to remediation by boiling the water to 100 °C for 30 min. This was done in order to kill the *Escherichia coli* that are possibly present which may hinder the remediation process, as well as the detection of heavy metals. Finally, 10 mg of each form of calamansi was added to 100 ml of the corresponding water sample and was left to stand for 5 days. The water samples were stirred for 10 min before and after the 5-day remediation. The remediated samples were then filtered twice through a filter membrane. After 0.1 M NaCl was added to each filtered sample, it was sonicated for 15 min. ASV analyses were performed to determine the Pb concentration in each water sample.

### Anodic stripping voltammetry

A three-electrode system was used for the ASV measurements. The modified carbon graphene electrode was used as the working electrode, the silver-silver chloride electrode as the reference electrode, and the bare carbon graphene electrode as the counter electrode. The electrolyte solution in the electrochemical cell was composed of 0.1 M NaCl and deionized water. Anodic stripping voltammetry was used for the detection of trace Pb. The electrodes were analyzed with a cleaning step at + 1 V for 60 s, a deposition potential of − 0.90 V for 90 s, an initial potential of − 0.90 V, a final potential of 0.90 V, a rest period for 60 s, and a scan rate of 0.05 V/s for the determination of the optimum amount of modifiers as well as the optimization of ASV Parameters.

## Results and discussion

### Determination of best modified SPGEs

**Figure 4:**
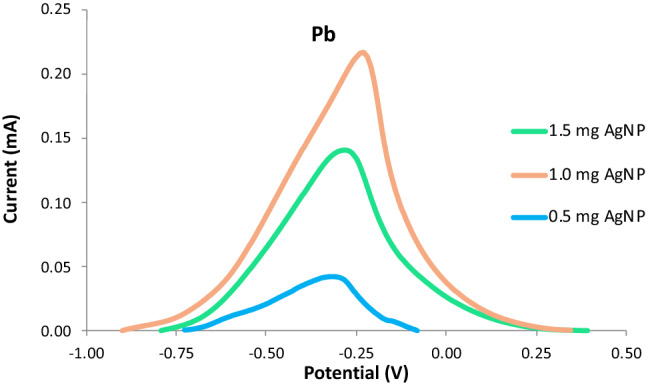
Voltammograms for varying amounts of electrode modifier.

**Figure 5:**
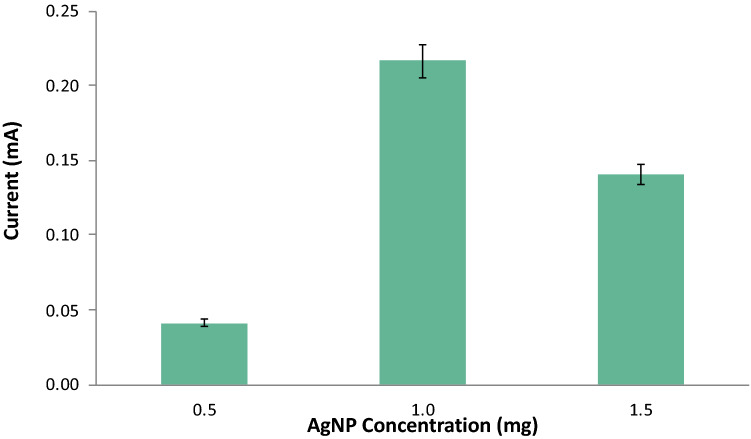
Anodic current peaks for varying amounts of AgNP.

The concentration of Bi was kept constant at 1.0 mg and the AgNP concentration was varied at 0.5 mg, 1.0 mg, and 1.5 mg. The Nafion-Ethanol solution was held constant at a mixture of 0.333 ml 1% (v/v) Nafion and 4.667 ml of pure ethanol. The resulting electrodes were used in the detection of 10 ppm of lead via anodic stripping voltammetry. The following ASV parameters were held constant: a cleaning step at + 1 V for 60 s, an initial potential of -0.90 V, a final potential of 0.90 V, deposition time of 90 s, a rest period for 60 s, and a scan rate of 0.05 V/s. The electrochemical measurements showed an increasing trend from the anodic current peak of Pb as the concentrations of AgNP concentrations were increased from 0.5 mg to 1.0 mg as seen in Figs. 4 and 5. However, a significant decrease in the anodic current peak was observed as the concentration reached 1.5 mg. The decrease in the response of the anodic current peaks can be ascribed to the oversaturation of the modifiers on the surface of the electrodes as the concentration of modifiers is increased. This created a thick layer of the modifiers on the surface of the working electrode inhibiting its conducting capabilities. Furthermore, as the peak increases the amount of analyte on the surface of the electrode also increases resulting in a much-needed higher potential to undergo reduction, thus, making the potential values shift slightly to the right as its peak increases. Since the fabricated electrode with 1.5 mg of AgNP exhibited the highest anodic current peak, it was determined to be the optimum amount of modifier that will exhibit the best measurements for the detection of Pb in the study.

### Optimization of ASV parameters

#### Deposition time

**Figure 6:**
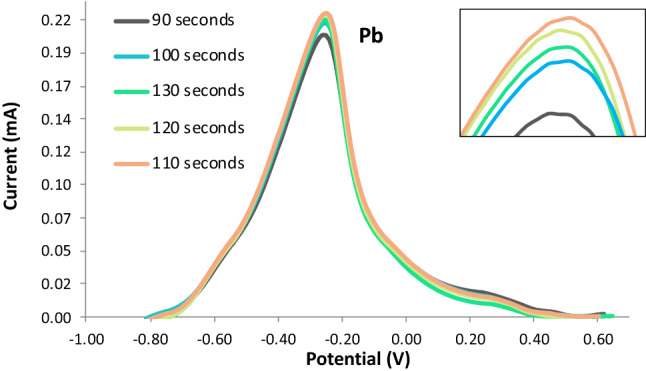
Voltammograms for varying deposition time.

**Figure 7:**
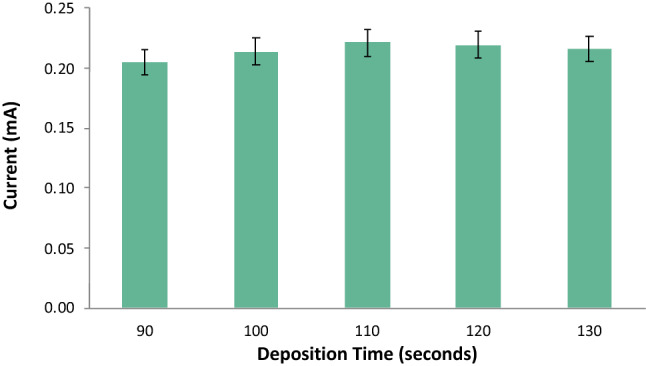
Anodic current peaks for varying deposition time.

The applied deposition time was varied at 90 s, 100 s, 110 s, 120 s, and 130 s. The other ASV parameters were held at constant with the initial potential at − 0.9 V, the final potential at 0.9 V, the rest period at 60 s, and the scan rate at 0.05 V/s. The voltammograms from the optimization of deposition time indicated an increased response of the current peak as the deposition time was increased from 90 to 110 s as seen in Figs. 6 and 7. However, it was observed that a deposition time of more than 120 s significantly decreased the current response peak. The decrease in the anodic current peak is caused by the huge amount of the analytes that oversaturated the electrode’s surface^36^. Therefore, the optimal deposition time was determined to be 110 s.

#### Initial potential

**Figure 8:**
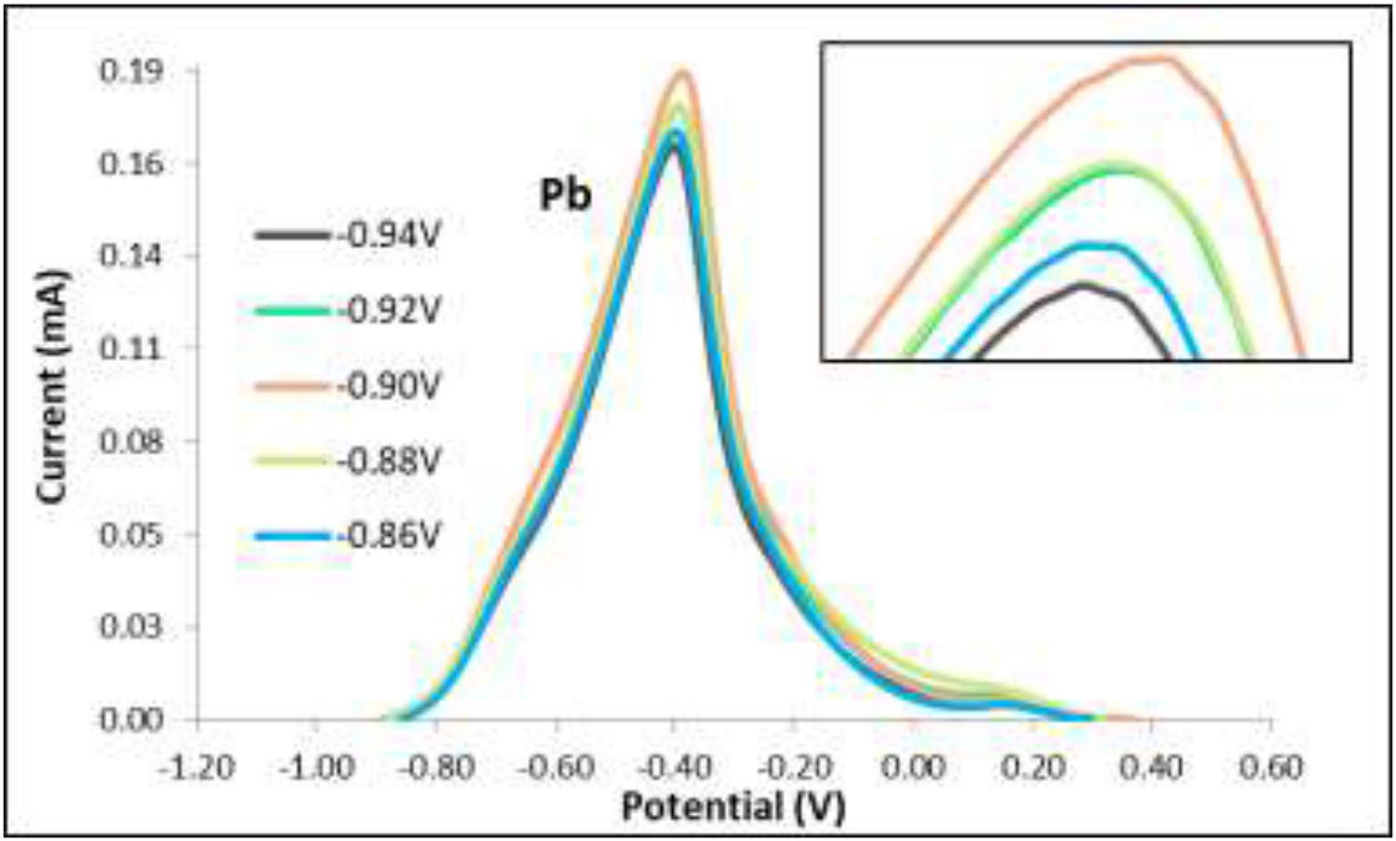
Voltammograms for varying initial potential.

**Figure 9:**
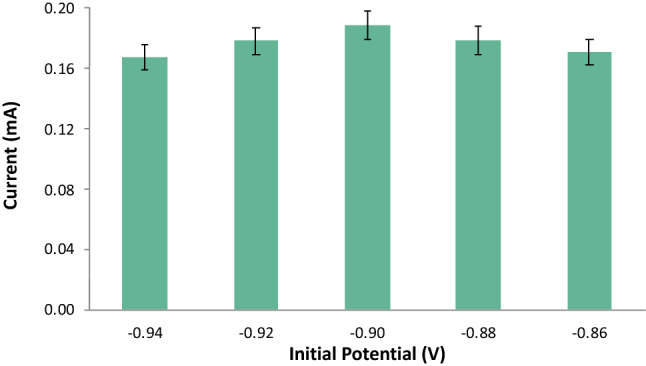
Anodic current peaks for varying initial potential.

The applied initial potential was varied at − 0.86 V, − 0.88 V, − 0.90 V, − 0.92 V, and − 0.94 V, and the other ASV parameters were held constant with the deposition time at 110 s was already the determined optimal time, the rest period at 60 s, and the scan rate at 0.05 V/s. An increasing trend of the anodic current response of Pb was observed as the initial potential was changed to a more negative value from − 0.86 V to − 0.90 V as seen in Figs. 8 and 9. Notwithstanding a noticeable decrease of the anodic current peak, the initial potential became a more negative potential than − 0.90 V. This was due to the evolution of hydrogen ions as the initial potential became more negative^37^. Therefore, the initial potential used in the study was determined to be − 0.90 V for the detection of Pb. This was deemed the optimum potential yielding the highest anodic current peak.

### Calibration curve

**Figure 10:**
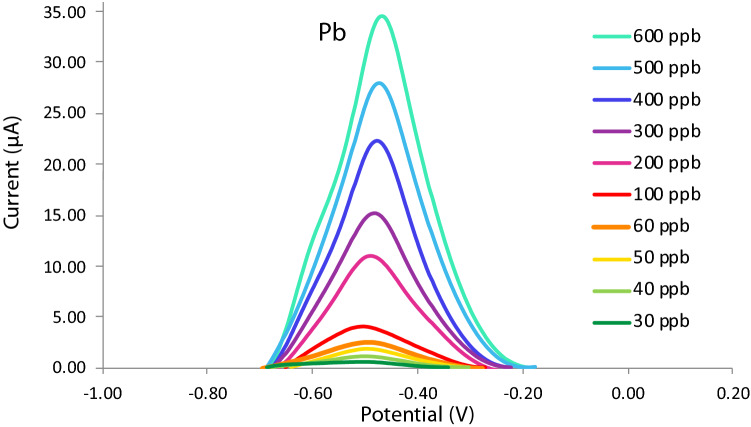
Voltammograms for varying Pb concentrations.

**Figure 11:**
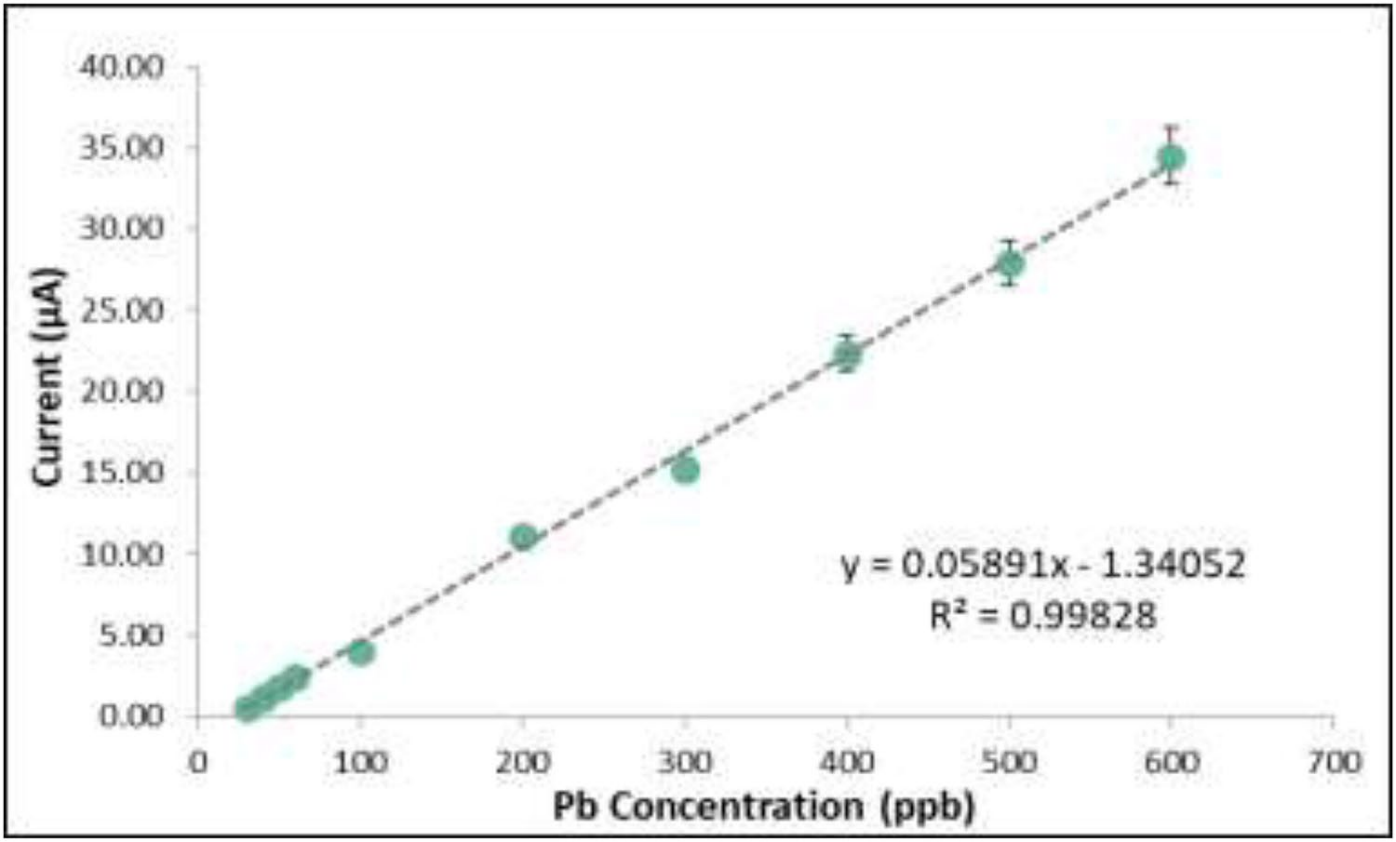
Calibration curve of the modified SPGE.

The concentration of Pb in the analyte solution was varied at 30 ppb, 40 ppb, 50 ppb, 60 ppb, 100 ppb, 200 ppb, 300 ppb, 400 ppb, and 500 ppb. Figure 10 shows the voltammetry curves for the said Pb concentrations. The current peak of each curve was plotted against its corresponding concentration to obtain the calibration curve. The linear equation obtained from this monotonic correlation was subsequently used to compute for the detected concentration of Pb in the Pasig River water samples as seen in Fig. 11. The values for R^2^ were calculated to be 0.99828 which signified a strong linear relationship between the concentration of Pb and the anodic current response.

### Characterization of the modified SPGE

#### Cyclic voltammetry

**Figure 12:**
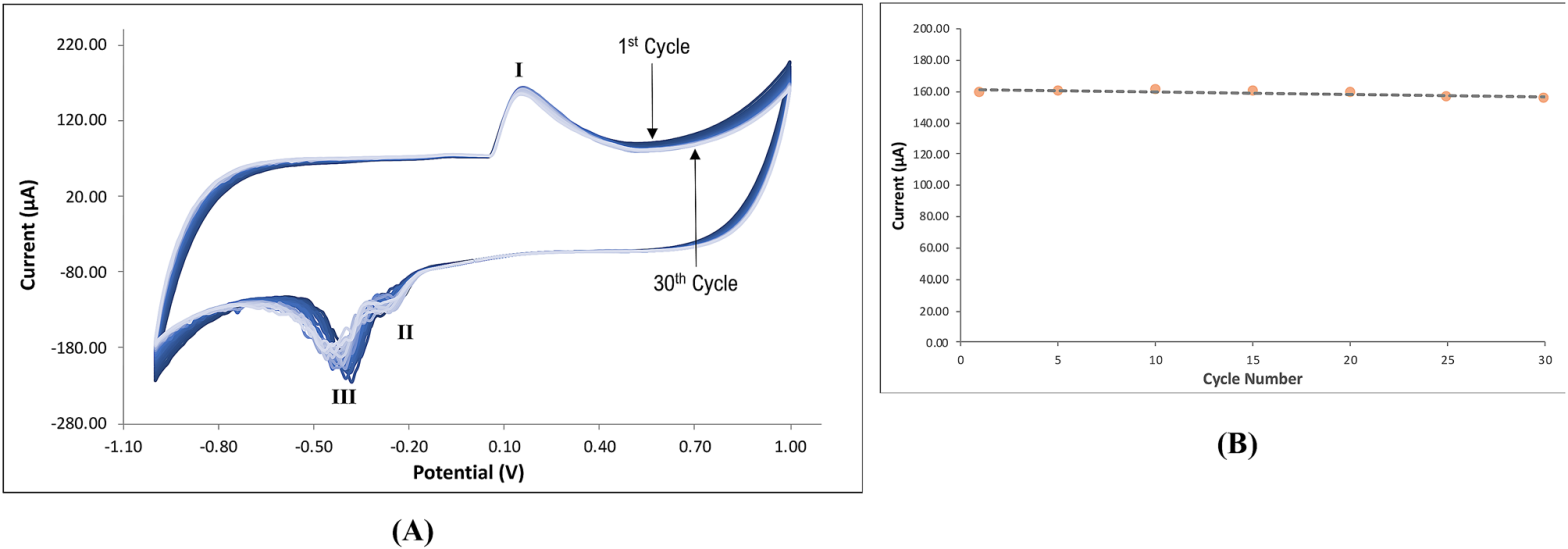
(**A**) Cyclic voltammogram of the modified SPGE for 30 cycles. (**B**) Peak I current vs. the number of cycles.

The stability of the modified SPGE was tested using cyclic voltammetry. Figure 12A shows the cyclic voltammetry curves of the modified SPGE for 30 cycles. Figure 12B shows the electrode’s excellent stability as the successive scans exhibited diminutive deterioration from the first up to the thirtieth scan. The forward scan from − 1.0 V to 1.0 V exhibited an anodic Peak I at the potential 0.15 V which corresponded to the combined peak of the reduction half-reaction of Bi^+^  + e^−^ → Bi and Ag^+^  + e^−^ → Ag^38^. On the other hand, the reverse scan from 1.0 V to − 1.0 V exhibited two cathodic peaks at the potential 0.25 V and 0.4 V. Due to agglomeration of AgNP, the cathodic peaks exhibited instability which can be seen in Fig. 12. The first cathodic Peak I refers to the oxidation half-reaction of Bi → Bi^+^  + e^−^ and Ag → Ag^+^  + e^−^. The second cathodic Peak II corresponds to further oxidation half-reaction of Ag^+^  → Ag^2+^  + e^−^^38^.

#### SEM and EDX analysis

**Figure 13:**
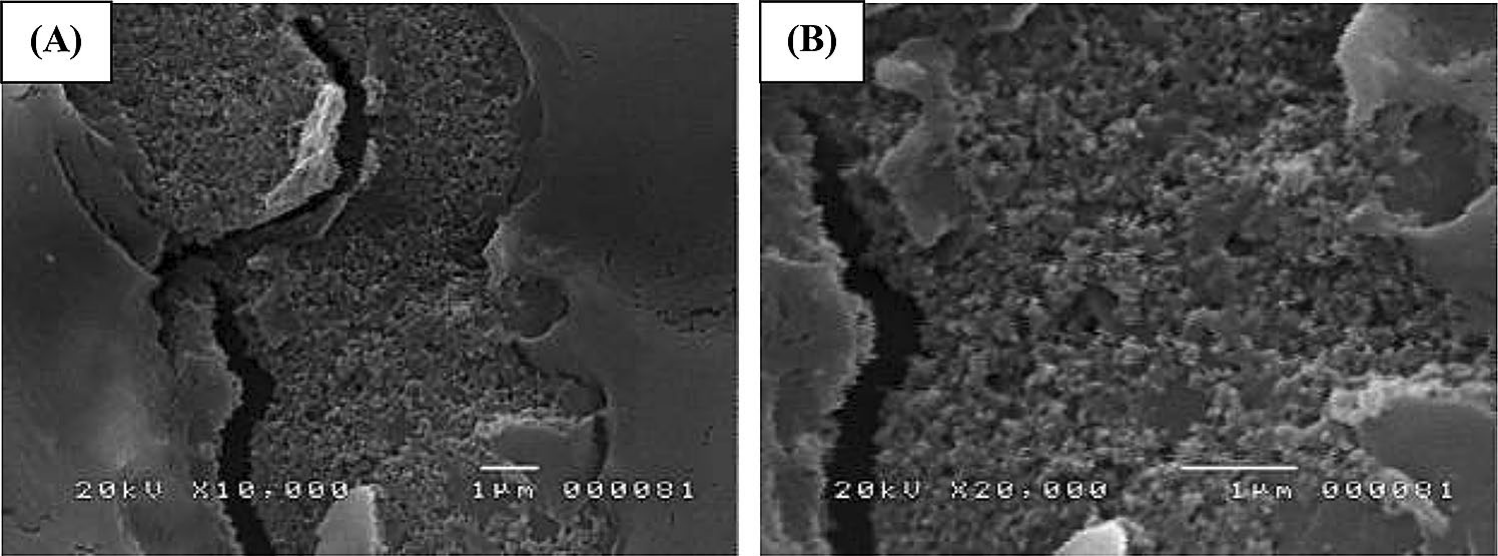
SEM micrographs of the modified SPGE at magnifications of (**A**) ×10,00 and (**B**) ×20,000 magnifications.

**Figure 14:**
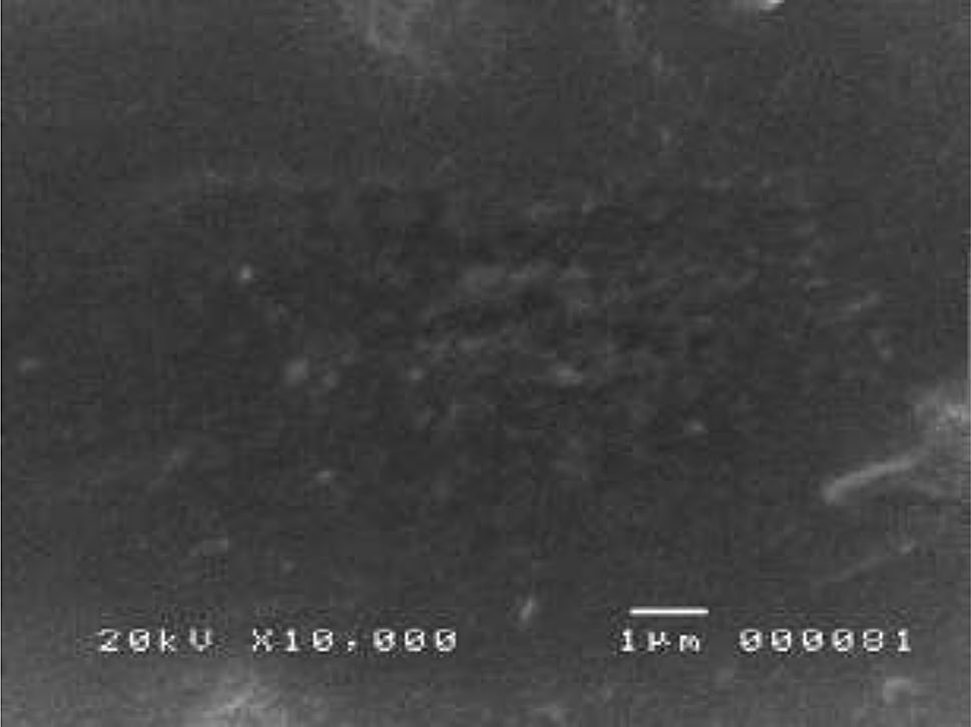
SEM micrograph of the bare SPGE at ×10,00 magnification.

**Figure 15:**
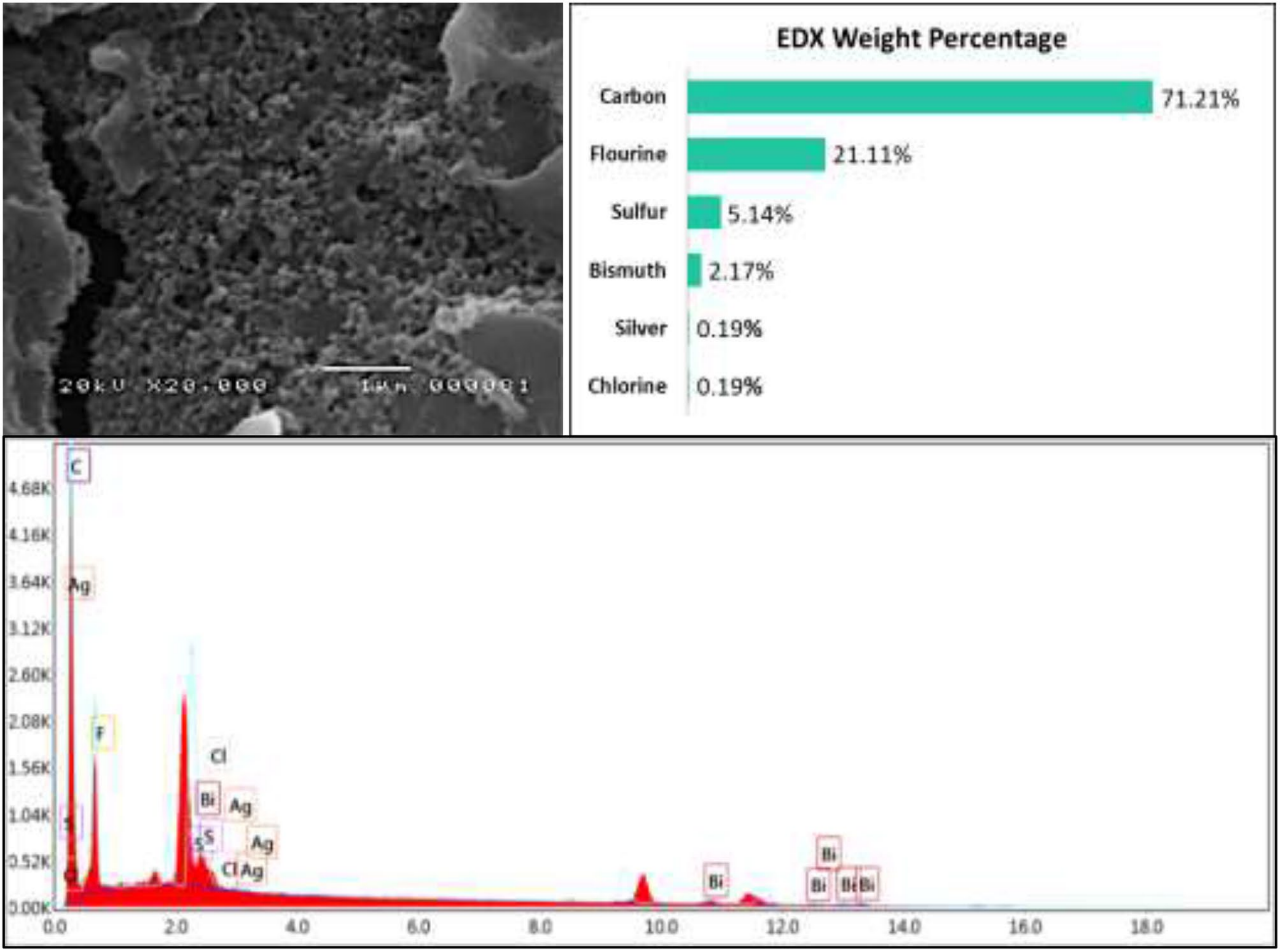
EDX profile of the modified SPGE.

The surface morphology analysis of the modified screen-printed graphene electrode modified with Bi and AgNP was done by taking a picture of the surface of the electrode prior to and after modification. Figure 13A,B show the successful deposition of Bi and AgNP on the electrode’s surface. For comparison, the surface morphology of the bare SPGE is shown in Fig. 14. The modified SPGE was also subjected to EDX analysis in order to confirm the elemental composition of the deposition on the surface of the electrode. Figure 15 shows the site of spectroscopy, the elemental composition in weight percentage, and the EDX spectrum taken at 20,000 magnification of the modified SPGE. The presence of Bi and Ag on a substrate of carbon is shown through EDX analysis which confirms the successful deposition of the modifiers onto the surface of the graphene electrode. The drop coating method of Bi modifiers promotes the electrode’s sensitivity by creating adsorption sites in which heavy metals attach to, thereby creating a larger current response and lowering the limit of detection^39^. The detected concentrations of fluorine and sulfur are associated with Nafion which acts as a binder for the modifiers and is also found to help improve an electrode’s sensitivity due to its unique structure of having a negatively charged skeleton^40^. The detected concentration of chlorine is attributed to the PVC medium in which the graphene electrode was screen printed onto. Although the PVC medium was fully covered with a layer of graphene electrode, the concentration of chlorine was also detected since the x-rays used in EDX spectroscopy are capable of giving information up to a depth of 1 µm from the surface of the electrode.

### Pasig River water samples remediation

#### Anodic stripping voltammetry

**Figure 16:**
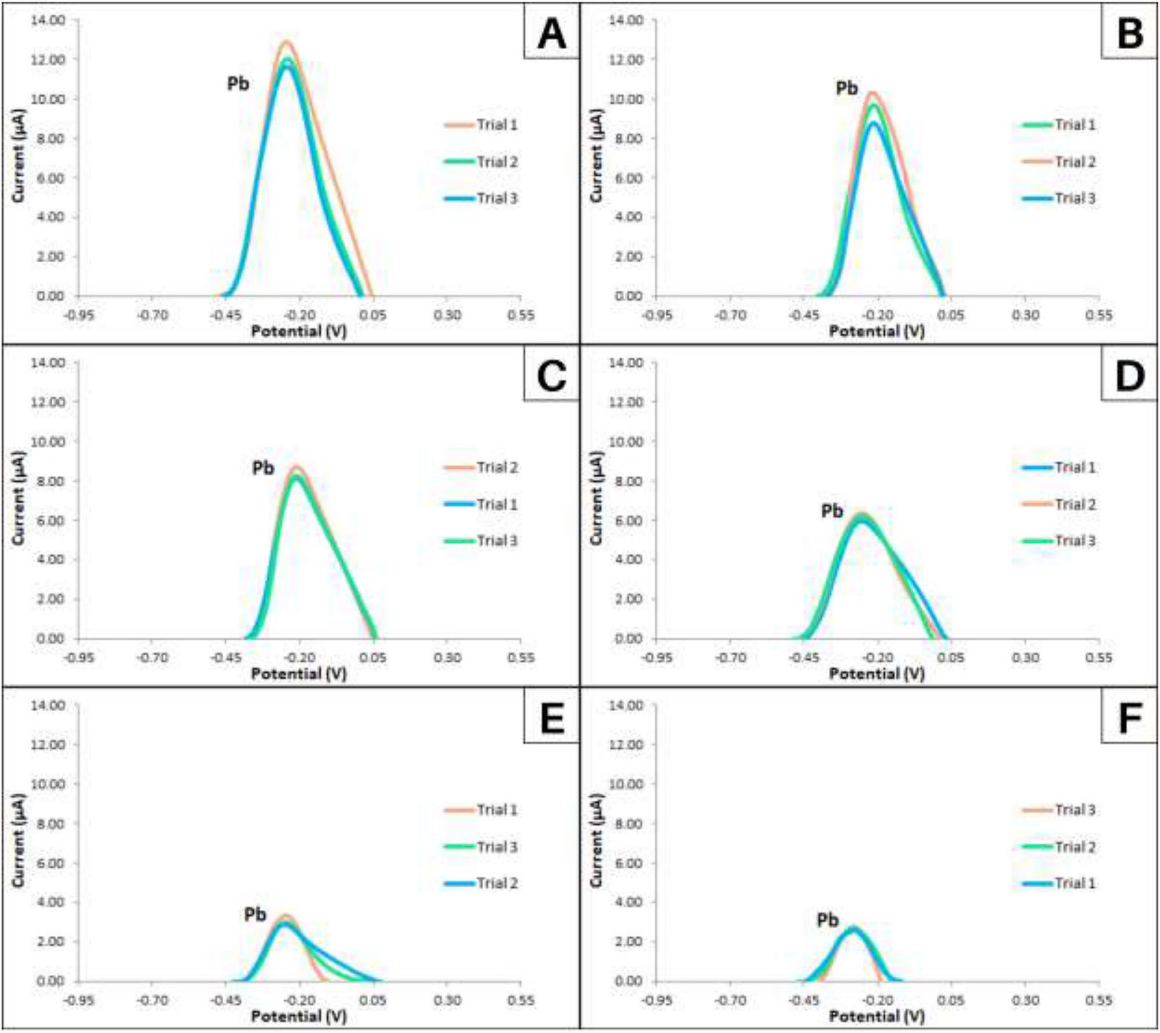
Voltammograms for detection of Pb in (**A**) unremediated water, (**B**) ground sun-dried calamansi powder remediated water, (**C**) dry-ashed calamansi remediated water, (**D**) food grade pectin remediated water, (**E**) fractionated pectin remediated water, and (**F**) AIS extracted pectin remediated water.

The determination of Pb concentrations in the water samples was done by using the optimized modified SPGE and optimum ASV parameters. Figure 16 shows the voltammograms of the detection of Pb in unremediated and calamansi*-* remediated water samples*.*

The voltammograms show that the fabricated Bi/AgNP/ Nafion screen-printed graphene electrodes were highly sensitive in detecting Pb in unremediated as well as remediated water samples. The remediation was done with sun-dried ground calamansi rinds*,* dry-ashed calamansi rinds, commercially available pectin, fractionated pectin, and AIS extracted pectin. Even the presence of 67.91 ± 9.6 ppb of Pb was detected in the AIS extracted pectin corresponding to approximately 2.5 µA anodic peak current was amply detected.

#### Atomic absorption spectroscopy

**Figure 17:**
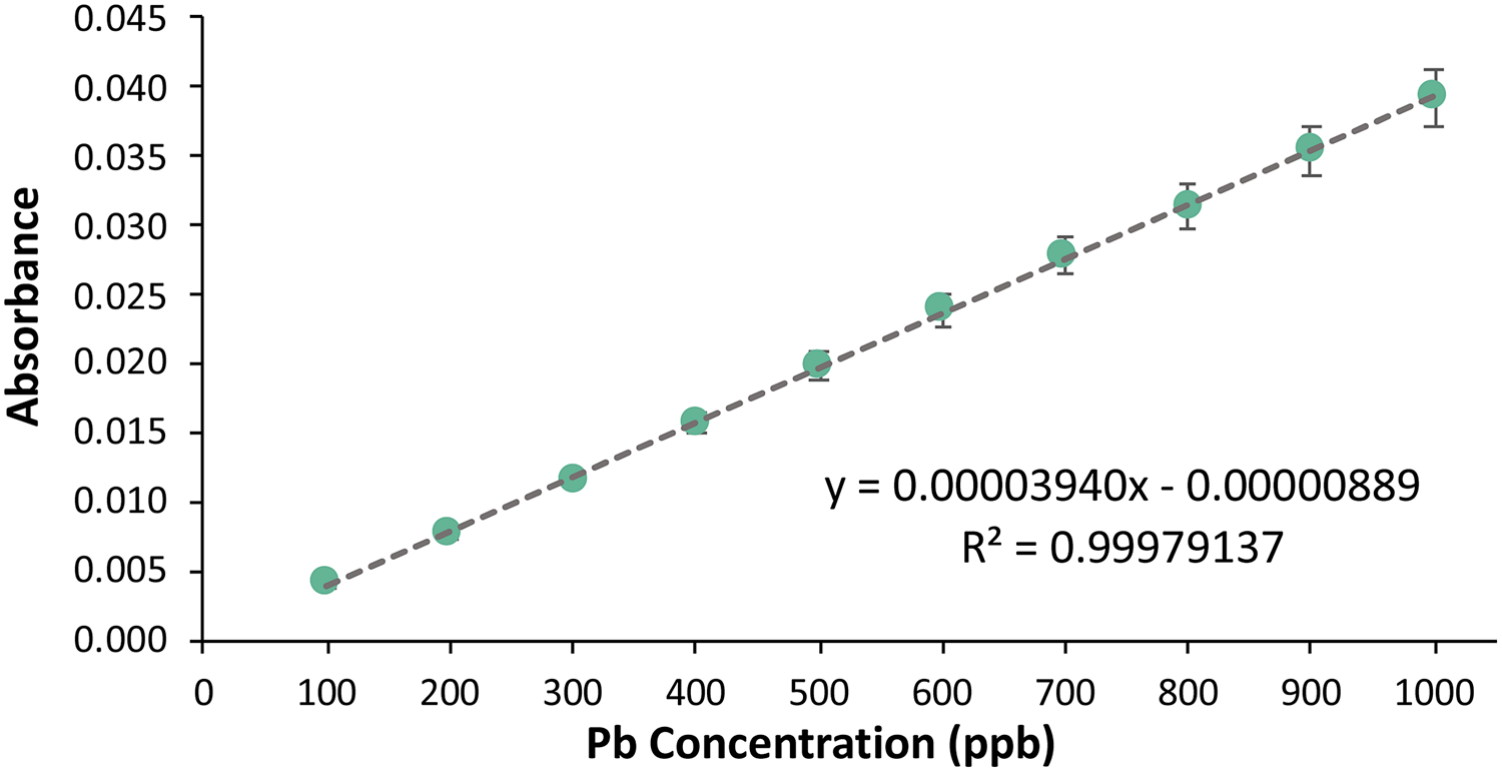
Pb calibration curve via AAS.

Determination of the Pb concentration in the Pasig River water samples via atomic absorption spectrometry was also performed to verify the results of the voltammograms. Figure 17 shows the calibration curve acquired from the detection of Pb in the range of 100 ppb to 1000 ppb via AAS. The concentrations of heavy metals in the real samples were determined by substituting the values of the absorbance as the y-values in the equations from the calibration curve of Cd and Pb.

#### Comparison of detected Pb concentration in ASV and AAS

The detected concentrations of Pb in the Pasig River water real samples from ASV and AAS method were compared through their calculated mean and standard deviation (Table 1). The detected concentrations of Pb from the voltammetric analysis were very similar to the results gathered from AAS which signifies that the fabricated modified screen-printed graphene electrode is as reliable as AAS.

**Table 1:** Comparison of detected Pb concentration in ASV and AAS.

Sample name	ASV (ppb)	AAS (ppb)	Percent difference (%)
Uremediated water	229.23 ± 10.81	227.81 ± 8.15	0.62
Ground calamansi rinds	185.44 ± 12.97	172.81 ± 6.71	7.05
Dry-ashed calamansi rinds	164.52 ± 5.38	166.89 ± 21.13	1.43
Food grade pectin	127.48 ± 3.39	112.75 ± 10.25	12.26
Fractionated pectin	74.68 ± 3.72	79.75 ± 20.35	6.57
AIS extracted pectin	68.36 ± 1.51	67.91 ± 9.6	0.66

#### Lead attenuation

**Figure 18:**
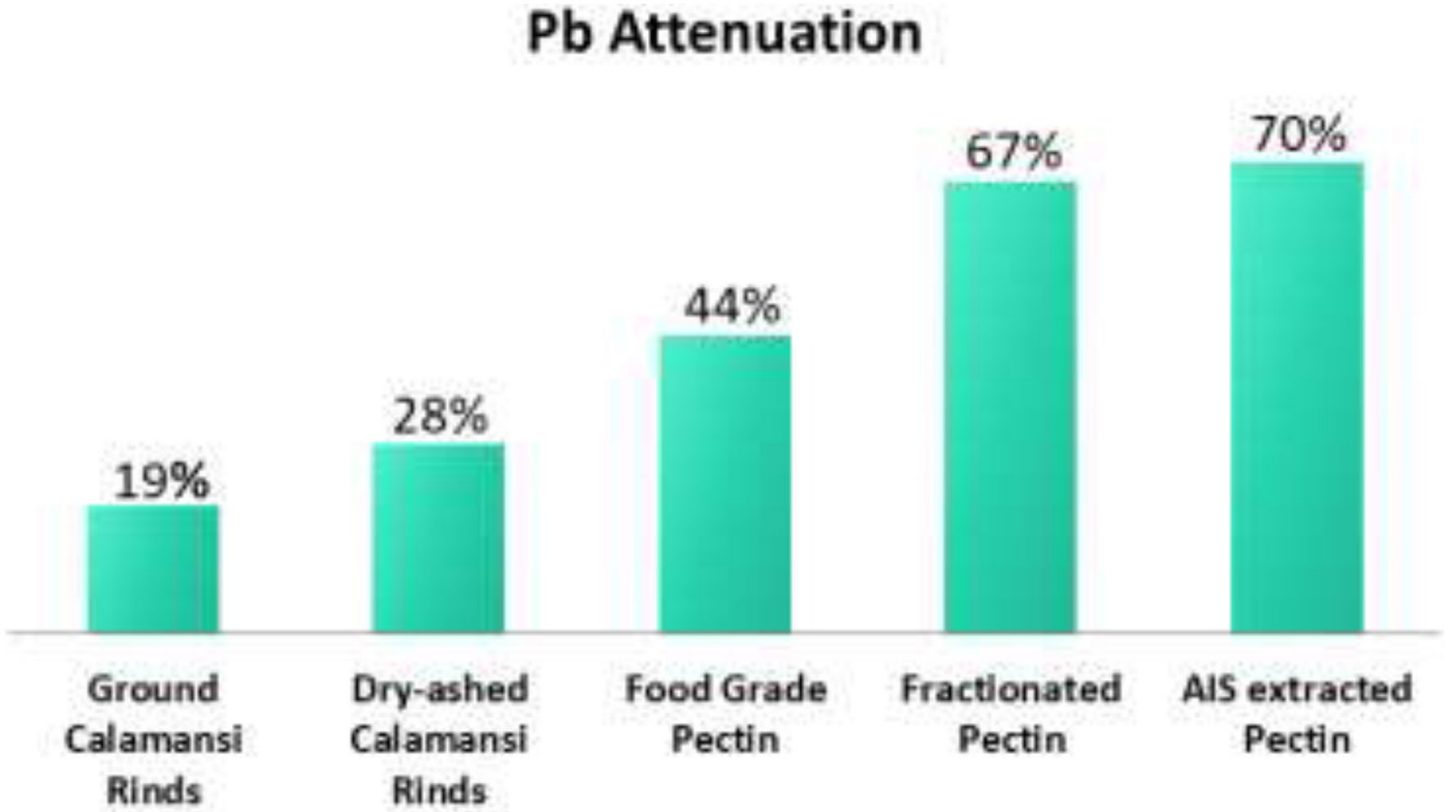
Lead attenuation of different forms of *calamansi rinds*.

From Fig. 18, it is evident that the best remediator was the AIS extracted pectin yielding a 70% lead attenuation, followed by fractionated pectin with 67% attenuation. Commercial food grade pectin was also effective with 44% attenuation. Dry-ashed calamansi rinds yielded 28% attenuation and even ground calamansi rinds which had only mechanical change provided 19% attenuation. These results show that indeed calamansi which is abundant in the Philippines is effective in removing Pb ions from the water making them cost-effective remediators even for economically challenged nations. This can effectively provide some solution to the availability of potable water to marginalized countries.

### Limit of detection and quantitation

The limit of detection (LOD) was calculated to be 267.6 ppt and a limit of quantitation (LOQ) of 810.8 ppt for Pb. The LOD of the modified screen-printed graphene electrode in this study was compared with similar electrodes from other literature as shown in Table 2. A thing to note in the table is the different types of methods used to modify the electrode, namely, electrodeposition and drop coating method. The former method is usually superior in properties because it results in a thin and uniform layer of the modifiers which also alloys to the electrode. However, this method requires a lot of long and complex processes while the drop coating method is very simple and easy to execute but often results in high detection limits. As can be seen in Table 2, electrodes modified through electrodeposition^41–43^ are more sensitive as compared to the ones modified through drop coating^44,45^. Despite this, our previous work^33^ and the fabricated electrode used in this study is at par with electrodes with electrodeposited modifiers. All things considered, the detection limits of our fabricated electrodes are superior when compared to other screen-printed electrodes.

**Table 2:** Performance comparison of the fabricated modified SPGE with other works.

Electrode	Modifiers	Coating method	Technique	Heavy metals detected	LOD for Pb	Ref
Screen-printed carbon electrode	Bismuth and chitosan	Electrodeposition	SWASV	Pb, Cd, and Zn	0.2 ppb	^41^
Screen-printed carbon electrode	RGO-MWNT-AuNP	Drop coating	SWASV	Pb and Cd	0.3 ppb	^44^
Screen-printed electrode	Single-walled carbon nanohorns	Drop coating	SWASV	Pb and Cd	0.4 ppb	^45^
Screen-printed expanded graphite electrode	Sb-SPAN	Electrodeposition	DPASV	Pb and Cd	0.2 ppb	^42^
Paraffin-impregnated graphite electrode	Poly methyl thymol blue	Electrodeposition	DPASV	Pb and Cd	0.18 ppb	^43^
Screen-printed graphene electrode	Bismuth and nafion	Drop coating	ASV	Pb and Cd	0.2 ppb	^33^
Screen-printed graphene electrode	Bismuth, silver nanoparticles, and nafion	Drop coating	ASV	Pb	0.2 ppb	This study

## Conclusions

A highly sensitive Bi/AgNP/Nafion-modified screen-printed graphene electrode was successfully fabricated for the detection of trace lead concentrations in river water samples. The optimum amount of silver nanoparticles was found to be 1.0 mg and the optimal deposition time and potential were found to be 110 s and − 9.0 V, respectively. Under these optimized conditions, the limit of detection of the sensor was found to be 267.6 ppt and a strong linear correlation (R^2^ = 0.999) between the Pb concentration and the anodic current response was obtained. The scanning electron micrographs and energy dispersive x-ray spectrum confirmed the presence of the modifiers on the electrode surface. Stability test showed insignificant depreciation even after 30 cycles. This sensor was used prior to and after a novel method of water remediation using calamansi rinds in different forms, viz., ground sun-dried, dry-ashed, food-grade pectin, fractionated pectin, and AIS—extracted pectin. All the forms of pectin remediated trace Pb concentration in varying amounts. The ground sun-dried calamansi rinds removed Pb concentration of up to 19% while the AIS-extracted pectin yielded the highest attenuation of up to 70%.

## Data Availability

The data sets generated during and/or analyzed during the current study are available from the corresponding author on reasonable request.
